# Enemy release from the effects of generalist granivores can facilitate *Bromus tectorum* invasion in the Great Basin Desert

**DOI:** 10.1002/ece3.5314

**Published:** 2019-07-17

**Authors:** Jacob E. Lucero, Urs Schaffner, Ghorbanali Asadi, Alireza Bagheri, Toshpulot Rajabov, Ragan M. Callaway

**Affiliations:** ^1^ Division of Biological Sciences and the Institute on Ecosystems The University of Montana Missoula Montana; ^2^ Department of Biology York University Toronto Ontario Canada; ^3^ CABI Switzerland Delémont Switzerland; ^4^ Department of Agronomy, College of Agriculture Ferdowsi University of Mashhad Mashhad Iran; ^5^ Department of Agronomy and Plant Breeding Razi University Kermanshah Iran; ^6^ Department of Botany and Plant Physiology Samarkand State University Samarkand Uzbekistan; ^7^ Wildlife Biology Program The University of Montana Missoula Montana

**Keywords:** biogeography, *Bromus tectorum* (cheatgrass), enemy release, generalist herbivory, invasion ecology, small mammals

## Abstract

The enemy release hypothesis (ERH) of plant invasion asserts that natural enemies limit populations of invasive plants more strongly in native ranges than in non‐native ranges. Despite considerable empirical attention, few studies have directly tested this idea, especially with respect to generalist herbivores. This knowledge gap is important because escaping the effects of generalists is a critical aspect of the ERH that may help explain successful plant invasions. Here, we used consumer exclosures and seed addition experiments to contrast the effects of granivorous rodents (an important guild of generalists) on the establishment of cheatgrass (*Bromus tectorum*) in western Asia, where cheatgrass is native, versus the Great Basin Desert, USA, where cheatgrass is exotic and highly invasive. Consistent with the ERH, rodent foraging reduced cheatgrass establishment by nearly 60% in western Asia but had no effect in the Great Basin. This main result corresponded with a region‐specific foraging pattern: rodents in the Great Basin but not western Asia generally avoided seeds from cheatgrass relative to seeds from native competitors. Our results suggest that enemy release from the effects of an important guild of generalists may contribute to the explosive success of cheatgrass in the Great Basin. These findings corroborate classic theory on enemy release and expand our understanding of how generalists can influence the trajectory of exotic plant invasions.

## 1 INTRODUCTION

One of the most widely cited explanations for the success of invasive plants in their non‐native ranges is the enemy release hypothesis (ERH), originally proposed by Elton (1958) but more explicitly formalized by Keane and Crawley (2002). The ERH asserts that the translocation of plant species across oceans or continents can geographically isolate them from natural enemies such as herbivores and pathogens, resulting in freedom from population controls imposed by these enemies. Such freedom could allow some exotic species to proliferate and become invasive in their non‐native ranges, where their new neighbors remain subject to population controls imposed by their natural enemies (Elton, 1958; Keane & Crawley, 2002). This idea can be tested by excluding natural enemies in both the native and non‐native ranges of an exotic invader to determine how enemies affect invader abundance in each range (Maron & Vila, 2001). The ERH predicts that natural enemies should limit invader abundance to a greater extent in the native range than in the non‐native range (Keane & Crawley, 2002; Maron & Vila, 2001).

Many empirical studies have addressed aspects of the ERH, but few have used exclusion treatments to quantify the effects of natural enemies on invader abundance in both the native and non‐native ranges. Instead, most studies have conducted biogeographic comparisons of enemy loads and inferred enemy release when fewer enemy species attacked invaders in the non‐native range compared with the native range (see review by Roy, Handley, Schonrogge, Poland, & Purse, 2011). Such results may demonstrate escape from natural enemies (e.g., Mitchell & Power, 2003), but they do not show release because reduced enemy loads may not translate to increased abundance for the invader (Beckstead & Parker, 2003). A salient example of “release” (sensu Keane & Crawley, 2002) is that of DeWalt, Denslow, and Ickes (2004). Using paired control and fungicide treatments in both the native (Costa Rica) and non‐native (Hawaii) ranges of invasive *Clidemia hirta*, they showed that fungicide applications increased the survival and relative growth rates of understory populations of *C. hirta* in the native range but not in the non‐native range. Thus, invasive populations of *C. hirta* in Hawaii had experienced some degree of enemy release from pathogenic fungi relative to native populations in Costa Rica. Other experiments have explicitly evaluated the effects of enemy exclusion in the native and non‐native ranges of invaders (Williams, Auge, & Maron, 2010), but such studies are relatively rare, an issue noted in several reviews (Liu & Stilling, 2006; Roy et al., 2011; Torchin & Mitchell, 2004).

Biogeographically explicit tests of enemy release are particularly scant in the context of generalist herbivores. Several experimental studies have explored aspects of enemy release from generalists (Halbritter, Carroll, Gusewell, & Roy, 2012; Joshi & Vrieling, 2005; Schaffner et al., 2011), but we know of none that have employed experimental exclosures in a biogeographic setting. This knowledge gap may stem from the notion that the effects of generalists are similar in both the native and non‐native ranges of exotic plants (Keane & Crawley, 2002). Because generalists consume many species, they are not necessarily restricted to the geographic distribution of any particular host species. Thus, translocated plants could potentially encounter generalists in any community. Indeed, native generalists in recipient communities often attack exotic plants (Morrison & Hay, 2011; Parker, Burkpile, & Hay, 2006; Parker & Hay, 2005; Pearson, Callaway, & Maron, 2011), which can result in population‐level suppression (i.e., biotic resistance; Pearson, Potter, & Maron, 2012; St. Clair, O'Connor, Gill, & McMillan, 2016). However, generalist herbivores do not always suppress populations of exotic plants (Connolly, Pearson, & Mack, 2014; Maron, Pearson, Potter, & Ortega, 2012; Orrock, Witter, & Reichman, 2008; Pearson et al., 2011). Thus, escape from the effects of generalists is an under‐studied component of the ERH, despite its theorized importance (Keane & Crawley, 2002).

*Bromus tectorum* (hereafter “cheatgrass”) invasion in the Great Basin Desert, USA, presents an opportunity to test the ERH in the context of generalist herbivores. Cheatgrass is an annual species native to Eurasia and northern Africa that was first noted in the Great Basin around the turn of the 20th century (Mack, 1981). Since then, cheatgrass has expanded to dominate at least 650,000 km^2^ of perennial grassland and shrubland in the central Great Basin (Balch, Bradley, D'Antonio, & Gomez‐Dans, 2013). Importantly, cheatgrass is much more abundant in North America than in Eurasia (Pearson et al., 2017). This biogeographic difference in abundance might be at least partially due to enemy release from generalists. Cheatgrass seeds in both the Great Basin and western Asia are vulnerable to predation by rodents—generalists that can strongly influence the identity and relative abundance of species in communities (Brown & Heske, 1990; Howe & Brown, 2000; Larios, Pearson, & Maron, 2017; Paine, Beck, & Terborgh, 2016; Sharp‐Bowman, McMillan, & St. Clair, 2017). However, several studies have shown that rodents in the Great Basin avoid cheatgrass seeds relative to seeds of native species (Kelrick, MacMahon, Parmenter, & Sisson, 1986; Lucero, Allen, & McMillan, 2015; Ostoja, Schupp, Durham, & Klinger, 2013), and rodent foraging can limit the establishment of native competitors to a greater extent than cheatgrass (Lucero & Callaway, 2018a, 2018b). Thus, cheatgrass in the Great Basin may disproportionately escape the effects of rodent foraging relative to native competitors. But testing whether such relative freedom constitutes enemy release requires experimental exclusion of rodents in both the native and non‐native ranges of cheatgrass (Keane & Crawley, 2002; Maron & Vila, 2001).

Our objective was to test the ERH by contrasting the effects of rodent foraging on cheatgrass establishment in the native versus non‐native range. To do this, we conducted parallel rodent exclosure and seed addition experiments in western Asia, where cheatgrass is native, and in the Great Basin, where cheatgrass is exotic and highly invasive. Examining consumer effects on cheatgrass establishment has important demographic implications because the population growth (λ) of cheatgrass depends strongly on establishment success (Griffith, 2010). To further explore the possibility that rodent interactions with cheatgrass seeds depend upon biogeographic context, we also examined the foraging preferences of rodents in western Asia and in the Great Basin with respect to seeds from cheatgrass and a suite of native competitors.

## 2 MATERIALS AND METHODS

### 2.1 Rodent effects on establishment

We examined rodent effects on cheatgrass establishment at four study sites in western Asia and five sites in the Great Basin. In western Asia, study sites were in the Razavi Khorasan (*n* = 2) and North Khorasan (*n* = 2) provinces of Iran. In the Great Basin, study sites were in Idaho (*n* = 1), Nevada (*n* = 3), and Utah (*n* = 1), USA. Locations of study sites are provided in Table A1. All study sites in Iran were separated by at least 20 km, and all sites in North America were separated by at least 80 km. These distances are orders of magnitude greater than individual rodents and annual grasses typically disperse over short time periods (Harper, Freeman, Ostler, & Klikoff, 1978; Hayssen, 1991; Jones, 1989; O'Farrell, 1978; Rehmeier, Kaufman, & Kaufman, 2004). Thus, study sites in each region sampled independent biological communities. All sites in both regions were located in communities dominated by native plants with <5% cover by invasive plants. Densities were low, but cheatgrass was present at each study site, suggesting that all sites were suitable for cheatgrass establishment. Rodent surveys suggest that native *Rattus* spp. and *Mus musculus* are the most abundant taxa near study sites in Iran (Sharif, Ziaei, Daryani, Nasrolahei, & Lackterashi, 2006), and no exotic rodent species have been reported in the area. In the Great Basin, rodent communities are dominated by native *Peromyscus maniculatus*, *Perognathus parvus*, *Dipodomys* spp., and *Tamias minimus* (Lucero et al., 2015, Phillips 2018). *Mus musculus* has been reported (Lucero et al., 2015), but this exotic species is rare (Phillips 2018).

At each site, we measured the effects of rodent foraging at seven sampling stations, each separated by 50 m. Each sampling station consisted of three exclosure treatments. In the first treatment, we sowed 100 cheatgrass seeds into a functional, “closed” exclosure that effectively excluded rodents. In the second treatment, we sowed 100 cheatgrass seeds into a nonfunctional, “open” exclosure that admitted rodents. In the third treatment, we installed a functional exclosure that excluded rodents but received no cheatgrass seeds. This third treatment served as a “control” to monitor cheatgrass recruitment from in situ seed banks. Closed and control exclosures were constructed of 1‐cm‐mesh hardware cloth assembled into 30 cm (diameter) × 30 cm (height) cylindrical cages with a floor and a roof. Floors and roofs prevented rodents from burrowing under or climbing into exclosures. Cages were installed by excavating 4 cm of topsoil with a garden hoe and then placing cages in the excavated pits. We secured cages into place by pounding 13‐cm (length) sod staples into the ground through cage floors with a hammer. We then replaced the excavated soil, except for large rocks and plant debris. Open cages were constructed and installed in a similar fashion, except for one 7 × 7 cm hole cut into the side of the cage at ground level to provide rodent access. We gently patted sown seeds ≈0.5 cm into the soil with the back of the hand. Burying seeds in this manner made them relatively inaccessible to invertebrate and avian granivores because only rodents locate buried seeds via olfaction (Kamil & Balda, 1985), and invertebrates do not dig for buried seeds (MacMahon, Mull, & Crist, 2000). It is possible that limiting invertebrate and avian access inflated establishment rates in experimental cages relative to wild (i.e., unmanipulated) communities, but we have no data to confirm this. Cheatgrass seeds were hand‐collected near study sites (see Table A1 for locations) during the summer of 2010 and 2014 in the Great Basin and Iran, respectively.

We installed exclusion experiments during August 2014 in both Iran and the Great Basin and left cages undisturbed until August 2015, when cheatgrass recruits were counted in all cages. Once counted, cheatgrass plants in the Great Basin were collected and destroyed to prevent the establishment of new populations. This protocol has successfully prevented cheatgrass invasion following other seed addition experiments in the Great Basin (Lucero et al., 2015).

To quantify the effects of rodent foraging on cheatgrass establishment, we contrasted the number of cheatgrass individuals established in open, closed, and control cages. We employed a linear mixed‐effects model using the lmer function in R (R Development Core Team, 2018) to analyze our data. This function calculates denominator degrees‐of‐freedom using Satterthwaite's method and compares multiple means using Tukey's method. We treated region (Iran vs. the Great Basin) and cage treatment (open vs. closed vs. control) as interacting fixed factors and study site within each region as a random factor. Treating study site as a random factor helped to account for any biologically relevant differences (e.g., rodent density, in situ germination rates, percent plant cover, elevation, temperature, precipitation) potentially present among study sites. We nested sampling station within study site. The ERH predicts that rodent foraging should limit cheatgrass establishment in Iran but not in the Great Basin. Specifically, in Iran, there should be fewer cheatgrass individuals established in open cages than in closed cages, but in the Great Basin, there should be a similar number of cheatgrass individuals established in open and closed cages, resulting in a significant region × treatment interaction.

### 2.2 Rodent seed preferences

Measuring region‐specific foraging preferences could enrich our mechanistic understanding of enemy release from generalists. Strictly speaking, region‐specific foraging preferences are not necessary for enemy release to occur. Exotic invaders could experience enemy release if natural enemies were absent or present at very low densities in the non‐native range relative to the native range (Keane & Crawley, 2002; Vermeij, Smith, Dailer, & Smith, 2009). However, evaluating region‐specific foraging preferences is potentially interesting in systems like ours where generalist consumers in both the native and non‐native ranges of the invader attack focal invaders and native competitors. Disproportionate escape from the effects of generalists in non‐native ranges (see Figure 1 in Keane & Crawley, 2002) could occur if generalists in native and non‐native communities had biogeographically distinct foraging preferences with respect to focal invaders, as reported by Schaffner et al. (2011). Thus, measuring the foraging preferences of rodents in both the native and non‐native ranges of cheatgrass could help explain the outcome of the exclusion experiments outlined above. However, we emphasize that enemy exclusion experiments, not preference experiments, test the central tenet of the ERH.

We evaluated the foraging preferences of rodents with respect to seeds from cheatgrass and a suite of native competitors using cafeteria‐style preference experiments. These experiments occurred at the same study sites used to measure rodent effects on cheatgrass establishment, with the addition of three sites in the Nurata District of Uzbekistan (*n* = 7 in western Asia, *n* = 5 in the Great Basin) to broaden our spatial scope of inference in the native range. Thus, except in Uzbekistan, our preference experiments potentially sampled the same rodents that drove the exclusion experiments outlined above. Locations of study sites for preference experiments are shown in Table A1.

At each site, we assessed the foraging preferences of rodents at seven sampling stations, each separated by 50 m. Each sampling station consisted of four feeding trays, constructed from 150 × 25 mm petri dishes, ¾‐filled with on‐site soil filtered through a 500‐μm sieve. The four trays were placed in a rectangular configuration on the ground roughly 7 cm apart from one another. One tray received 3 g of seed from cheatgrass, and each of the other trays (designated as “native trays”) received 3 g of seed from a different, locally common, native grass. Seeds in all trays were thoroughly incorporated into the filtered soil to protect them from invertebrates and birds (Kamil & Balda, 1985; MacMahon et al., 2000).

Seed selection by rodents can depend on seed mass, as rodents often prefer large seeds to small ones (Maron et al., 2012; Pearson et al., 2011; Reader, 1993). To account for this, seeds in the first native tray weighed more than those of cheatgrass, seeds in the second native tray weighed less, and seeds in the third native tray weighed approximately the same. In Iran, we replaced the “small‐seeded” species with a large‐seeded species, and in Uzbekistan, the “similar‐sized” species was larger than cheatgrass. These deviations occurred because we could not find species with ideal seed sizes near the study sites. Table A2 presents the species offered to rodents at each site, the weights of their individual seeds, and how seeds were procured.

We left seed trays undisturbed in the field for 72 consecutive hours, after which they were collected and processed. Data collection ended on 15 October 2013 in Iran; 22 October 2013 in Uzbekistan; and 17 October 2015 in the Great Basin. We recovered seeds remaining in feeding trays by passing the trays' contents (filtered soil, debris introduced by foraging rodents, and remaining seeds) through the same 500‐μm sieve mentioned above, through which filtered soil passed easily but not seeds. We removed dirt and/or organic debris from recovered seeds and then weighed the sample to the nearest 0.01 g. We subtracted this weight from the original 3 g to determine the mass of seeds removed by rodents. We log‐transformed our data to improve normality. We assumed that seed preference and seed removal were positively related such that few remaining seeds indicated high preference.

To analyze the seed preferences of granivorous rodents, we employed three linear mixed‐effects models (one for Iran, one for Uzbekistan, and one for the USA) using the lmer function in R (R Development Core Team, 2018). Within each country, we treated species identity (i.e., seed mass) as a fixed factor and study site as a random factor. We analyzed each country separately because species identity varied among countries. We did not use linear regressions to explore our data because we offered only four species to rodents in each country.

If patterns of seed preferences are consistent with predictions based on seed mass and the ERH, seed removal and seed mass should be positively related only for native species in each region (Pearson, Ortega, Eren, & Hierro, 2018). Specifically, in Iran and Uzbekistan, rodents should remove seeds from native species, including cheatgrass, according to seed mass such that cheatgrass seeds do not disproportionately escape removal relative to other native competitors. In the Great Basin, however, rodents should remove seeds from native species but not cheatgrass according to seed mass such that cheatgrass seeds disproportionately escape removal relative to native competitors (Pearson et al., 2018).

## 3 RESULTS

### 3.1 Rodent effects on establishment

**Figure 1:**
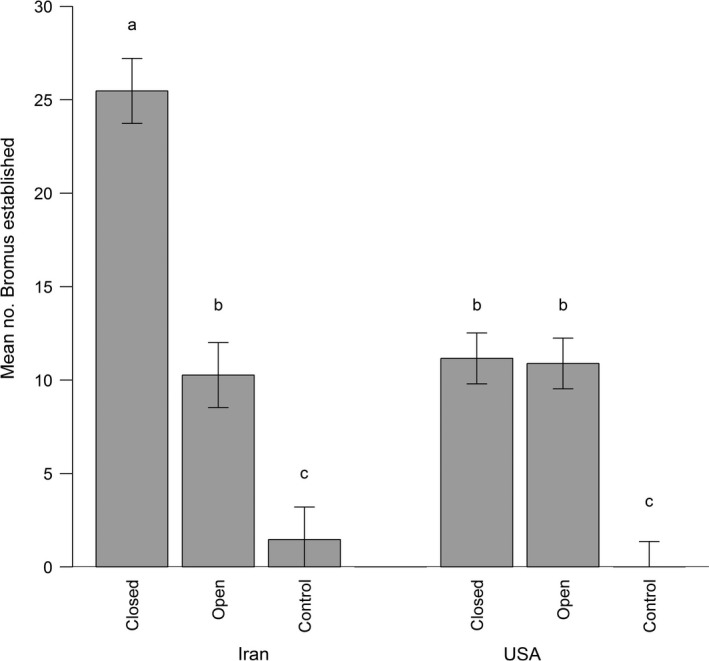
Mean (±*SE*) number of cheatgrass (*Bromus tectorum*) individuals established in closed (protected from rodents, seeds added), open (exposed to rodents, seeds added), and control (protected from rodents, no seeds added) cages in Iran and the Great Basin Desert, USA. Differences in establishment between closed and open cages within a region are due to rodent foraging, and establishment from control cages is due to seed banks. Means that do not share letters differ significantly (*p* < 0.05)

Our analysis of rodent effects on cheatgrass establishment revealed a significant main effect of region (Iran vs. the Great Basin; *F_1,3.35_* = 11.98, *p* = 0.03), a significant main effect of treatment (open vs. closed vs. control cages; *F_2,60.00_* = 155.96, *p* ≪ 0.01), and a significant region × treatment interaction (*F_2,60.00_* = 33.02, *p* ≪ 0.01). Most importantly, rodent foraging reduced cheatgrass establishment in Iran but not in the Great Basin (Figure 1). In Iran, 25.63 ± 1.56 *SE* cheatgrass individuals established in closed cages that received seeds but only 10.43 ± 1.56 individuals established in open cages that received seeds (*df* = 60.00, *t‐*ratio = 9.46, *p* < 0.01), a difference of 59.31%. In the Great Basin, however, cheatgrass recruited 11.23 ± 1.15 *SE* individuals in closed cages received seeds and 10.96 ± 1.15 individuals in open cages that received seeds (*df* = 60.00, *t*‐ratio = 0.25, *p* = 0.99). Thus, rodent effects on cheatgrass establishment were region‐specific. Interestingly, cheatgrass did not recruit appreciably from seed banks in either Iran or the Great Basin (Figure 1). On average, 2.63 ± 1.56 *SE* individuals recruited per control cage that received no seed additions in Iran, and 0.05 ± 1.15 individuals recruited per control cage that received no seed additions in the Great Basin. These means did not significantly differ (*df* = 7.43, *t*‐ratio = 1.33, *p* = 0.76), and the 95% confidence intervals of both means (2.63 ± 3.036 in Iran, 0.05 ± 2.25 in the Great Basin) included zero, suggesting that recruitment from seed banks was negligible in both countries and therefore unlikely to affect our estimates of rodent effects.

We observed a region‐specific bias in cheatgrass establishment in rodent‐free cages that received seeds. More cheatgrass seedlings established in closed cages that received seeds in Iran (25.63 ± 1.56 *SE*) than in the Great Basin (11.23 ± 1.15 *SE*; *df* = 7.43, *t*‐ratio = 7.42, *p* < 0.01). Importantly, however, neither of these means had 95% confidence intervals that included zero (25.63 ± 3.06 for Iran and 11.23 ± 2.25 in the Great Basin).

### 3.2 Rodent seed preference

**Figure 2:**
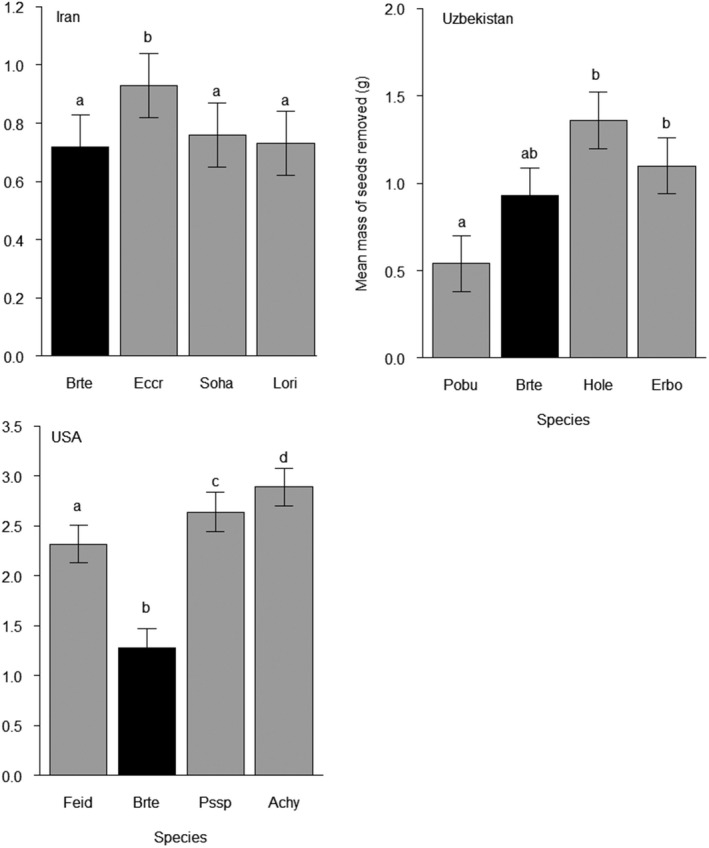
Mean (±*SE*) mass (g) of seeds removed by rodents in Iran, Uzbekistan, and the Great Basin Desert, USA during cafeteria‐style preference experiments. Bars for cheatgrass (*Bromus tectorum*) are colored black for emphasis. Species are arranged along *x*‐axes in ascending order of seed mass (see Table A2 for species names and seed masses). Means that do not share letters differ significantly (*p* < 0.05). Note different scales on *y*‐axes

Region‐specific patterns of seed removal by rodents generally followed predictions derived from seed mass and the ERH (Figure 2). In Iran, we found a significant main effect of species identity on seed removal (*F*_3,204.03_ = 5.20, *p* < 0.01). Specifically, rodents removed 0.72 ± 0.11 *SE* g of cheatgrass, 0.93 ± 0.11 g *Echinochloa crus‐galli*, 0.76 ± 0.11 g of *Sorghum halepense,* and 0.73 ± 0.11 g of *Lolium rigidum* (see Table A3 for all pairwise contrasts). Importantly, interspecific variation in seed mass was relatively low among these species (0.7 mg; Table A2). Thus, cheatgrass seeds did not disproportionately escape removal relative to other native species in Iran, with the exception of similar‐sized *E. crus‐galli* (Figure 2, Table A3).

In Uzbekistan, we found a significant main effect of species identity on seed removal (*F*_3,54.00_ = 6.62, *p* < 0.01). Specifically, rodents removed 0.54 ± 0.16 *SE* g of *Poa bulbosa*, 0.93 ± 0.16 g of cheatgrass, 1.4 ± 0.16 g of *Hordeum leporinum,* and 1.1 ± 0.16 g of *Eremopyrum bonaepartis* (see Table A3 for all pairwise contrasts). Thus, rodents preferred the relatively large seeds of *H. leporinum* and *E. bonaepartis* to the relatively small seeds of *p. bulbosa*. Interestingly, no species was removed at a significantly higher rate than cheatgrass, suggesting that cheatgrass seeds did not disproportionately escape removal relative to other native competitors in Uzbekistan (Figure 2, Table A3).

In the Great Basin, we also found a significant main effect of species identity on seed removal (*F*_3,108.12_ = 47.22, *p* < 0.01). Specifically, rodents removed 2.32 ± 0.19 *SE* g of *Festuca idahoensis*, 1.28 ± 0.19 g of cheatgrass, 2.64 ± 0.20 g of *Pseudoroegneria spicata,* and 2.89 ± 0.19 g of *Achnetherum hymenoides* (see Table A3 for all pairwise contrasts). Thus, rodents in the Great Basin removed native seeds but not cheatgrass seeds as expected based on seed mass, and cheatgrass seeds were removed at a lower rate than any native species, regardless of mass. Even the relatively diminutive seeds of *F. idahoensis* were removed at almost twice the rate of cheatgrass seeds. Thus, cheatgrass seeds disproportionately escaped removal relative to all native competitors in the Great Basin (Figure 2, Table A3).

## 4 DISCUSSION

Relatively few studies have measured the effects of natural enemies—especially generalist herbivores—on populations of invasive plants in both native and non‐native communities. Here, we found that rodents, an important guild of generalist consumers, reduced cheatgrass establishment by approximately 60% in western Asia, where cheatgrass is native, but had no effect in the Great Basin, where cheatgrass is exotic and highly invasive (Figure 1). Interestingly, our main finding corresponded with a region‐specific foraging pattern: rodents selected against cheatgrass seeds relative to seeds from native competitors in the Great Basin but not in western Asia (with the exception of one species in Iran; Figure 2). Our results suggest that enemy release from the effects of rodent foraging—possibly due to region‐specific seed preferences—might help explain why cheatgrass is much more abundant in North America than in western Asia (Pearson et al., 2017).

Our study coincides with recent reports that rodent foraging in intact (i.e., undisturbed) Great Basin communities has little effect on cheatgrass populations. In a long‐term study conducted in Utah, USA, St. Clair et al. (2016) showed that rodent exclusion did not affect cheatgrass density in an undisturbed shrub‐steppe community. Lucero and Callaway (2018b) extended this work by showing that rodent foraging reduced the establishment of multiple species of more‐preferred (Lucero et al., 2015) native grasses by >80% each but had no effect on cheatgrass across an ≈80,000 km^2^ portion of the Great Basin (see also Lucero & Callaway, 2018a). Combined with the biogeographic perspective of the present study, these findings strongly suggest that enemy release from the effects of rodent foraging can promote cheatgrass invasion in the Great Basin.

It is not clear why rodents reduced cheatgrass establishment in western Asia but not the Great Basin. One potential explanation is that seed loss due to rodent foraging was relatively minor in the Great Basin compared with western Asia. To test this, we contrasted the total biomass of seeds removed (all species combined) by rodents during preference experiments in Iran, Uzbekistan, and the Great Basin using a linear mixed‐effects model with region (Iran vs. Uzbekistan vs. Great Basin) as a fixed factor and site within each region as a random factor. If seed loss due to rodent foraging was minor in the Great Basin compared with western Asia, the total biomass of seeds removed in the Great Basin should have been significantly lower than in Iran or Uzbekistan. This was not the case. Rodents removed 0.78 g (±0.22 *SE*) of seed per tray in Iran, 0.98 g (±0.19) in Uzbekistan, and 2.28 g (±0.14) in the Great Basin (*df* > 6.60, *t‐*ratio> *|*5.50|, *p* < 0.01 for both Iran–Great Basin and Uzbekistan–Great Basin pairwise comparisons). Thus, rodents in the Great Basin removed over twice as much seed from feeding trays as rodents in either Iran or Uzbekistan, suggesting that seed loss due to rodent foraging was more, not less, intense in the Great Basin than western Asia at the temporal, spatial, and taxonomic scale of our study.

Alternatively, evolutionary naivety may leave native rodents in the Great Basin relatively under‐equipped to exploit cheatgrass seeds. It has long been appreciated that plants and herbivores can evolve in response to one another (Ehrlich & Raven, 1964; Janz, 2011).  In this context, herbivores—including generalists like rodents—may be well equipped or behaviorally inclined to exploit the plant species with which they evolved, but under‐equipped or behaviorally adverse to exploit exotic plants with unfamiliar traits (van Kleunen, Weber, & Fischer, 2010; Schaffner et al., 2011). For example, Cappuccino and Carpenter (2005) suggested that some invasive plant species in northeastern North America may disproportionately escape herbivory because they possess biogeographically novel phytochemicals (i.e., “novel weapons”; Callaway & Aschehoug, 2000) that render them unpalatable to native consumers. Importantly, Great Basin rodent communities are comprised primarily of native genera that share no evolutionary history with cheatgrass (e.g., Lucero et al., 2015, Philips, 2018). However, the extent that novel weapons influence the feeding preferences of generalist vertebrates (e.g., Kalisz, Spigler, & Horvitz, 2014) in invaded communities is generally unclear. Alternatively, native rodents in the Great Basin could avoid cheatgrass seeds relative to seeds from native competitors due to inferior nutritional quality (Kelrick & MacMahon, 1985) and/or effective physical defenses (e.g., persistent awns) (Ceradini & Chalfoun, 2017).

The biogeographic germination bias we observed between western Asia and the Great Basin did not drive our main finding. Establishment from closed cages that received seeds was greater than zero in both Iran and the Great Basin (mean establishment in closed cages in Iran = 25.63 ± 1.56 *SE* individuals; mean establishment in closed cages in the Great Basin = 10.43 ± 1.56 individuals), indicating that rodents in both ranges could have imposed detectable effects on cheatgrass establishment, but only rodents in Iran actually did so. Biogeographic germination biases may have been due to the different ages of seeds used in Iran versus the Great Basin. Although cheatgrass seeds can remain viable for over 11 years in storage (Hulbert, 1955), older seeds may not germinate as readily as fresh seeds (Rice & Dyer, 2001). Alternatively, seed pathogens such as *Pyrenophoroa semeniperda* may have reduced cheatgrass establishment in the Great Basin but not in western Asia. *Pyrenophoroa semeniperda* can impose high mortality on cheatgrass seeds in the Great Basin (Beckstead, Meyer, Molder, & Smith, 2007), but this pathogen is extremely uncommon in Eurasia (Stewart, Allen, & Meyer, 2009) and has never been reported in Iran (Yonow, Kriticos, & Medd, 2004).

Our estimates of rodent effects on cheatgrass establishment did not account for the potential role of granivorous rodents as seed dispersers. Rodent species found near our study sites in Iran (e.g., Williams, Karl, Bannister, & Lee, 2000) and the Great Basin (e.g., Hollander & Vander Wall, 2004) can remove seeds from one location and cache them elsewhere without consuming them (Vander Wall, Kuhn, & Beck, 2005). In some cases, seed caching by rodents can improve plant establishment and bolster population growth (Longland, Jenkins, Vander Wall, Veech, & Pyare, 2001). However, this may not be the case for cheatgrass in the Great Basin. McMurray, Jenkins, and Longland (1997) showed that rodent caching may decrease cheatgrass establishment by increasing intraspecific competition among seedlings. Thus, it is possible that we underestimated the effects of rodent foraging in the Great Basin, but we have no data to confirm this. Beyond this paper, the effects of rodent foraging—including seed caching—on cheatgrass establishment in the native range have not been explored.

Our preference experiments reinforce the idea that rodent interactions with cheatgrass seeds depend upon biogeographic context. Based on seed mass, we found that cheatgrass seeds disproportionately escaped removal relative to seeds from native competitors in the Great Basin, but not in western Asia, with the exception of *E. crus‐galli* in Iran (Figure 2). In this case, it could be argued that *E. crus‐galli* was “over‐preferred,” as rodents removed its seeds at a higher rate than any other species, and seeds of all other species were removed at the same rate (Table A3). Regardless, our preference results add to a growing consensus that rodents in the Great Basin generally prefer seeds from native species to seeds from cheatgrass (Kelrick et al., 1986; Lucero et al., 2015; Ostoja et al., 2013). Importantly, we re‐emphasize that our rodent exclusion, not preference experiments, tested the ERH.

Biogeographic differences in the effects of rodent foraging probably cannot fully explain the success of cheatgrass in the Great Basin. Many factors operating at multiple temporal and spatial scales can influence the success of cheatgrass and other invasive plants in their non‐native ranges, including disturbance regimes, feedbacks with the abiotic environment, and biotic interactions with native species (Blackburn et al., 2011; Catford, Jansson, & Nilsson, 2009; D'Antonio & Vitousek, 1992; Mitchell et al., 2006). These factors are not mutually exclusive, and some may contribute more to invasion success than others under different conditions (Williams et al., 2010). That said, our study affirms Keane and Crawley's (2002) fundamental assertion that enemy release from the effects of generalists can contribute to the success of invasive plants in non‐native communities.

## CONFLICTS OF INTEREST

None declared.

## AUTHOR CONTRIBUTIONS

Conceived the study: JEL. Designed the study: JEL, RMC, and US. Conducted the experiments: JEL, GA, AB, and TR. Analyzed the data: JEL and RMC. Wrote the manuscript: JEL and RMC.

## Data Availability

Data are archived in the Dryad repository, https://doi.org/10.5061/dryad.rv70cs1.

## APPENDIX A

**Table A1:** Locations of study sites used to determine effects (“E”) of rodent foraging on cheatgrass (*Bromus tectorum*) establishment and rodent foraging preferences (“P”) with respect to seeds from cheatgrass and native competitors

Region	Country	Nearest town	GPS coordinates	Experiments conducted
Western Asia	Iran	Shirvan	37°23′46.15″N, 58°11′37.15″E	E, P
		Shirvan	37°38′34.13″N, 57°39′24.38″E	E, P
		Mashhad	36°12′57.33″N, 60°4′2.26″E	E, P
		Mashhad	36°3′30.41″N, 59°39′8.79″E	E, P
	Uzbekistan	Nurota	40°41′14.82″N, 65°36′36.16″E	P
		Nurota	41°4′35.52″N, 63°0′9.86″E	P
		Nurota	43°25′47.98″N, 64°37′27.36″E	P
North America	USA	Challis	44°12′8.65″N, 113°56′9.88″W	E, P
		Jackpot	41°55′28.70″N, 114°43′44.96″W	E, P
		McGill	39°58′26.51″N, 114°40′10.10″W	E, P
		Baker	39°1′6.34″N , 114°25′53.44″W	E, P
		Vernon	40°6′54.99″N , 112°32′4.37″W	E, P

**Table A2:** Species offered to rodents during preference trials, the mass (±95% CI) of their respective seeds (per seed), and how seeds were procured

Region	Country	Species offered	Seed mass (mg)	Mode of accession
Western Asia	Iran	*Bromus tectorum*	3.1 (0.1)	Hand‐collected
		*Echinochloa crus‐galli*	3.2 (0.1)	Hand‐collected
		*Lolium rigidum*	3.8 (0.1)	Hand‐collected
		*Sorghum halepense*	3.8 (0.1)	Hand‐collected
	Uzbekistan	*Bromus tectorum*	3.1 (0.1)	Hand‐collected
		*Eremopyrum bonaepartis*	4.2 (0.1)	Hand‐collected
		*Hordeum leporinum*	3.9 (0.1)	Hand‐collected
		*Poa bulbosa*	1.3 (0.1)	Hand‐collected
North America	USA	*Achnatherum hymenoides*	3.9 (0.1)	Purchased
		*Bromus tectorum*	3.1 (0.1)	Hand‐collected
		*Festuca idahoensis*	1.4 (0.1)	Purchased
		*Pseudoroegneria spicata*	3.2 (0.1)	Purchased

Note

“Hand‐collected” seeds were gathered by the authors near study sites (see Table A1 for coordinates). “Purchased” seeds were field‐collected and distributed by Granite Seed Co.

**Table A3:** Species‐level pairwise contrasts of seeds removed by rodents in Iran, Uzbekistan, and the USA during cafeteria‐xmlstyle preference experiments

Country	Contrast	*df*	*t*‐ratio	*p*‐value
Iran	Brte—Eccr	204.1	3.40	0.01
	Brte—Lori	204.1	0.18	0.99
	Brte—Soha	204.1	0.44	0.97
	Eccr—Lori	204.0	−3.31	0.01
	Eccr—Soha	204.0	−3.06	0.01
	Lori—Soha	204.0	0.27	0.99
Uzbekistan	Brte—Erbo	54.0	0.99	0.76
	Brte—Hole	54.0	2.31	0.11
	Brte—Pobu	54.0	−2.02	0.20
	Erbo—Hole	54.0	1.31	0.56
	Erbo—Pobu	54.0	−3.01	0.02
	Hole—Pobu	54.0	−4.32	<0.01
USA	Achy—Pssp	108.3	−3.06	0.01
	Brte—Feid	108.0	4.23	<0.01
	Brte—Achy	108.1	11.36	<0.01
	Brte—Pssp	108.1	7.72	<0.01
	Feid—Achy	108.2	6.79	<0.01
	Feid—Pssp	108.1	3.49	<0.01

Note

See Table A2 for species names.
